# Psychological Factors and Alcohol Use in Problematic Mobile Phone Use in the Spanish Population

**DOI:** 10.3389/fpsyt.2017.00011

**Published:** 2017-02-03

**Authors:** José De-Sola, Hernán Talledo, Gabriel Rubio, Fernando Rodríguez de Fonseca

**Affiliations:** ^1^Faculty of Psychology, Department of Psychobiology, Complutense University of Madrid (Universidad Complutense de Madrid), Madrid, Spain; ^2^St. Ignatius of Loyola University (Universidad San Ignacio de Loyola), Lima, Peru; ^3^Department of Psychiatry, Hospital Universitario 12 de Octubre, Madrid, Spain; ^4^Mental Health Clinical Management Unit, Institute of Biomedical Research in Malaga, University Regional Hospital of Malaga (Instituto de Investigación Biomédica de Málaga – IBIMA, Hospital Regional Universitario de Málaga), Malaga, Spain; ^5^Hospital Universitario 12 de Octubre, Madrid, Spain

**Keywords:** mobile phone addiction, problematic mobile phone use, mobile phone overuse, factors in mobile phone addiction, alcohol, tobacco, mobile phone use

## Abstract

This research aims to study the existing relationships among the factors of state anxiety, depression, impulsivity, and alcohol consumption regarding problematic mobile phone use, as assessed by the Mobile Phone Problem Use Scale. The study was conducted among 1,126 participants recruited among the general Spanish population, aged 16–65 years, by assessing the predictive value of these variables regarding this problematic use. Initially tobacco use was also considered being subsequently refused because of the low internal consistency of the scale used. In general terms, the results show that this problematic use is mainly related to state anxiety and impulsivity, through the dimensions of Positive and Negative Urgency. Considering its predictive value, multiple regression analysis reveals that state anxiety, positive and negative urgency, and alcohol consumption may predict problematic mobile phone use, ruling out the influence of depression.

## Introduction

There have been many and various attempts to find the key determinants of mobile phone addiction or problematic use, coupled with discussions on whether it is an addiction, as with substances, or a behavior that, in situations of abuse, may lead to similar issues. It is clear that mobile phones, as with many technology-related behaviors, foster situations of problematic use, especially among young people and adolescents, although such situations are also found in adult populations (1).

There is a logical and known coexistence between substance use and behavioral addictions (2–6). However, objectively, over and above this debate, there are far-reaching consequences that are associated with problematic mobile phone use, such as insomnia and sleep disorders (7–11), stiffness and muscular problems, eye problems (12), pain and weakness in thumbs and wrists (13), auditory and tactile illusions (14, 15), anxiety and mood swings (16, 17), high blood pressure (18), and behavioral and social problems such as “sexting” or the impulse to send or receive pictures or videos possessing sexual content (19, 20), mobile phone use in hazardous or prohibited situations, and the widespread interference of mobile phone use in personal, professional, social, or family life (21–23).

Against this backdrop, the need to find variables that are associated with or those that determine dependence and problematic mobile phone use has led to research on factors, such as education, occupation, lifestyle, gender, age, personality, and drug use. They are all interrelated and have been analyzed as precursors, mediators, or consequences of abusive behavior, and it is not always easy to find direct relationships that explain their causality.

Most likely one of the most relevant personality traits in behavioral addictions is impulsivity (24), a multifactorial trait that can be defined as a predisposition to quickly and immediately react without premeditation to internal or external stimuli, without considering the damage and negative consequences (25). In other words, it is a behavior that occurs with little or no precaution (26) and that involves significant cognitive distortions (27, 28). In this regard, several studies have found a relationship between impulsivity and addiction, dependence, or problematic mobile phone use (21, 29–31).

In general, as with other personality factors, impulsivity is considered to be a precursor or mediator or to coexist with addictions and psychopathological behaviors (32–35). Thus, its relationship with alcohol use has been observed (36–39), though this relationship has not always been evident without the mediation of intermediate variables such as certain psychopathological traits (40). Nor is it easy to find studies that show a direct relationship between alcohol abuse and problematic behavior due to mobile phones, given that, in many cases, there is the intermediation of factors such as depression (41–43).

The relationship between problematic mobile phone use and depression has also been investigated, although historically, research in the context of the Internet has predominated. In principle, there are differences between the psychopathological manifestations of problematic mobile phone use and the Internet because the latter is more characteristic of introverts and loners (44). Thus, depression may more frequently coexist with Internet abuse and anxiety with problematic mobile phone use (45). However, anxiety and depression are often linked, which has led to studies in which both may be predictors, which is a means of coping with personal dysphoria and may also lead to sleep disturbances (46), an aspect shared with the Internet (8).

Similarly, there is a clear relationship between problematic mobile phone use and anxiety (17, 45, 47), in addition to their coexistence with insomnia and depression, as noted above (7, 9). Specifically, Lepp et al. (48) find that mobile phone use is positively related to anxiety and that anxiety has a negative relationship with the level of personal life satisfaction. This may lead to the concept of social anxiety, already noted by Merlo (49), which is related to the need for the device, in addition to impulsivity and urgency in regard to sending and responding to messages (11, 16, 17). In general, social anxiety (50, 51) and environmental dependence (52–54) may have close relationships with mobile phone dependence, more frequently among women because of their greater sensitivity to interpersonal relationships, mainly through interactive social applications (55).

Finally, tobacco use and its relationship to problematic mobile phone use has also been investigated, although this coexistence has not always been demonstrated, as in the case of Jenaro et al. (9), who find no significant relationships. By contrast, Sánchez-Martínez and Otero (56), Toda et al. (57), and López-Fernández et al. (58) confirm this relationship. As in other cases, it is important to consider the possibility of intermediate personality variables, such as extraversion or self-esteem (47).

This research is based on the search for relationships among anxiety (expressed as state anxiety), depression, impulsivity, and alcohol use regarding problematic mobile phone use in the Spanish population. However, based on historical outcomes, we hypothesize that only anxiety and impulsiveness may be psychological determinants and predictors of problematic mobile phone use, which is also related to depression and alcohol use that may be circumstantial both as a cause and an effect or simply may coexist with such problematic use.

Initially tobacco use was also considered in this research, being refused because of the low internal consistency of the scale used. However, cannabis, psychostimulants, or other type of illegal drugs were not finally considered because of the lightweight in the context of a general population. A focused study of problematic phone use in patients diagnosed of illegal drug use disorders has to be considered for a future separate study in order to clarify the potential existence of mutually interacting factors in this in specific segment of the population.

The study extends beyond adolescents, covering the entire Spanish population. We believe that mobile phones, as an element of dependence and problematic use, have ended up capturing large segments of the adult population, becoming an addiction due to the similarity of its manifestations with the criteria of substance abuse and pathological gambling.

## Materials and Methods

The research project was examined and approved by the Ethics Committee of the Regional Hospital of Málaga-IBIMA institute.

### Sample and Participants

The sample includes 1,126 respondents from a survey of 1,600 questionnaires at the national level, both men and women, with an age range of 16–65 years. The survey procedures automatically exclude and erase uncompleted questionnaires, so only full respondents were used. The sampling was performed by using a non-probability procedure by quotas proportionate to the size of the Spanish population in the 17 Autonomous Communities, except Ceuta and Melilla, according to data from the National Statistics Institute (Instituto Nacional de Estadística) in 2014. Slightly more than half of the interviews were conducted in provincial capitals and in cities of over 100,000 inhabitants and the rest in rural areas and small towns (Table 1).

**Table 1:** **Distribution of the sample with respect to geographic area, age, gender, main occupation, and level of schooling**.

**Autonomous communities**	
Andalucía	15.7%
Aragón	2.5%
Asturias	2.0%
Balearic islands	1.9%
Canary islands	3.9%
Cantabria	1.2%
Castilla La Mancha	3.9%
Castilla León	4.4%
Catalonia	13.1%
Extremadura	2.3%
Galicia	5.0%
La Rioja	0.8%
Madrid	26.2%
Murcia	2.5%
Navarra	1.1%
Basque country	3.5%
Valencia	10.0%
**Age**	
16–25 years	40.9%
26–35 years	24.0%
36–45 years	17.0%
46–55 years	13.1%
56–65 years	5.0%
**Gender**	
Male	47.7%
Female	53.3%
**Main occupation**	
Worker	57.3%
Unemployed	20.2%
Student	18.7%
Household duties	3.8%
**Schooling**	
Higher education	63.5%
Middle education	30.4%
Basic education	6.1%
**Parents educational level**	
Higher education	28.4%
Middle education	27.0%
Basic education	40.4%
No schooling	4.2%
**Illegal drugs use**	
Cannabis and/or psychostimulants	5.5%
**Legal drugs use**	
Alcohol use ever	43.5%
AUDIT > 8	20.1%
Tobacco use ever	19.6%
Fagerström > 4	7.5%

To obtain information comparable to other studies, quotas were also set by age, with the over-representation of segments of between 16 and 25 years and between 26 and 35 years. The sample average is 32.8 years, with a SD of 11.67, with 47.7% being male and 53.3% female. Regarding occupation, more than half of the respondents work, and the rest are unemployed, students, and people who take care of household duties. The level of education is high, with a majority corresponding to higher education or university degrees; almost a third completed secondary school, whereas a minority does not have education beyond basic or elementary school. The educational level of parents mainly corresponds to basic studies, followed by higher education and secondary education, and a small share of parents have no schooling. With respect to tobacco use, and using the Fagerstrom scale, we identified 85 cases with moderate problematic use (score > 4). Regarding alcohol use, 286 cases had a moderate risk of alcohol-induced harm with AUDIT scores > 8 (Table 1). Finally, 5.5% of the sample use illegal drugs, basically cannabis (77 cases) and psychostimulants (13 cases).

### Procedure

The research was conducted through an online questionnaire that was sent between January and December 2014. Emailed links allowed each participant to access a platform from which the interview would begin through the survey software SSI Web version 6.8 by Sawtooth Software. It could be stopped to go back to the interview when necessary, and this link was disabled once the questionnaire was completed. All participants had to have their own mobile phone, which was assessed using a first initial filter question.

Approximately 20% of the sample was obtained through emails sent by us. The remaining questionnaires were completed by an online survey and sociological research company that used its database of 151,170 people in Spain, finally ending up with 1,126 answered questionnaires.

### Statistical Analysis

The data analysis was performed using SPSS v.23.

That analysis included Pearson correlations, first, between the total scores of the scales of state anxiety (STAI-S), depression (BDI-13), alcohol use (AUDIT), impulsivity in its five dimensions and total (UPPS-P), and problematic mobile phone use [Mobile Phone Problem Use Scale (MPPUS)] for each of its three factors and total. Second, the Pearson correlations of alcohol use and depression were specifically obtained over the other variables except MPPUS.

Subsequently, multiple regression analyses were conducted using the “Intro” system of SPSS, which simultaneously introduces all variables without removal, considering the total score of the MPPUS and the scores of each of its three factors separately as dependent variables, to ascertain the predictive value both globally and for each of the components. As independent variables, we included finally the state anxiety, alcohol use, depression scores, and the scores of the five dimensions of the UPPS-P separately (positive urgency, negative urgency, lack of premeditation, lack of perseverance, and sensation seeking), omitting in this case total impulsivity as a variable to avoid redundancy with the UPPS-P dimensions.

In all cases, the maximum level of significance admitted was 5%.

### Instruments

Mobile phone problematic use, state anxiety, depression, impulsivity, and alcohol use were assessed through the MPPUS (47), the UPPS-P Impulsive Behavior Scale (37, 59–61), the Alcohol Use Disorders Identification Test (AUDIT), the Beck Depression Inventory (BDI-13) (62), and the State Anxiety Inventory Scale (STAI-S) (63).

Initially, we also considered in the analysis tobacco consumption, using the Fagerström Test for Nicotine Dependence (FTND) (64, 65), being later discarded for further analysis due to its low internal consistency.

### Mobile Phone Problematic Use Scale

In assessing problematic mobile phone use, the MPPUS was used (47), with our adaptation of the MPPUS to the Spanish adult population, in turn based on the work of López-Fernández et al. (58) among adolescents (MPPUSA).

In that research, an exploratory factor analysis provided four factors or components that explain 59.8% of the variance. The first factor, with 25.9% of the variance, called “Abuse and Dependence,” is defined by excessive mobile phone use and recurrent thoughts, mood swings when the phone cannot be used, problems and interference in everyday life, discomfort and personal awareness of abuse, or warnings from the social environment. The second factor, comprising 17.6% of the variance, called “Craving and Loss of Control,” considers problems that arise from the progressive abandonment of activities, incapacity of control, or as a resource to compensate for dysphoric moods. The third factor, which entails 12.3% of the variance, called “Social Environment Dependence,” involves the personal perception of mobile phone dependence in relevant social environments. The last factor, with a single item (“I never have enough time for mobile phones”) and an explained variance of 4%, may define the tendency toward a progression or increase in the use of the device. This factor was not used because of its limited utility and practical statistical significance.

Therefore, this research analyzes problematic use, both from the total score of the MPPUS and separately, by considering these first three factors.

### Impulsivity: The UPPS-P Impulsive Behaviour Scale

When measuring impulsivity, we used the UPPS Impulsive Behavior Scale (61) in its latest five-dimension version (UPPS-P) (37, 59, 60) and Spanish adaptation by Verdejo-García et al. (66).

It consists of 59 items with Likert-type scales ranging from 1 to 4, depending on the level of agreement. It has five dimensions: negative urgency, which expresses the tendency to experience strong impulses under conditions of negative or dysphoric affective states; positive urgency, or the tendency to act hastily in response to positive emotional states; lack of premeditation, characterized by the lack of reflection or anticipation prior to the consequences of the behaviors themselves; lack of perseverance, or the difficulty in focusing on a task even though it is long, difficult, or boring; and sensation seeking, which may include both the tendency to seek and enjoy exciting activities and openness to new experiences, although in some cases they can be dangerous, thus having positive and negative aspects (67). Some of the items are written in reverse, which is an aspect that was corrected in the statistical analyses.

### Depression: The Beck Depression Inventory (BDI-13)

For depression, we used the BDI (68) in its 13-item reduced version (BDI-13) (63).

It considers affective, cognitive, motivational, and physiological symptoms of depression. The 13 items, with Likert-type scales, have four answer choices ranging from 0 to 3 points, with a maximum of 39 points.

### State Anxiety: The State-Trait Anxiety Inventory (STAI-S)

State anxiety was assessed using the STAI (69) by considering the Spanish adaptation of the STAI-S (63). State anxiety versus trait anxiety refers to transitional moments or periods characterized by tension, apprehension, and increased activity of the autonomic nervous system, which can vary in time or intensity. We have used state anxiety versus trait anxiety because it refers to the present time, in principle more objectifiable, assuming that state anxiety may also be reflected in trait anxiety.

The STAI-S has 20 items with Likert-type scales ranging from 0 to 3 and a possible range of 0 to 60 points; some items are written in reverse, which was corrected in the final statistical analyses.

### Alcohol Use: The Alcohol Use Disorders Identification Test (AUDIT)

Alcohol use was evaluated using the AUDIT. It consists of 10 closed-ended questions: the first eight have five response options ranging from 0 to 4, whereas the last two have three options, with a possible score of 0, 2, and 4. They measure the frequency and amount of alcohol use, dependence, and problems derived from its use.

## Results

### Reliability and Internal Consistency of the Instruments

Except for the tobacco use (FTND), finally excluded, and alcohol use (AUDIT), in general terms the instruments used in this research showed adequate internal consistency coefficients through Cronbach’s alpha, in line with those obtained in other studies.

Thus, in this study, the MPPUS presents an alpha of 0.939, with a mean score of 68.95 and an SD of 36.89 (Table 2). This consistency is similar to other studies in which coefficients between 0.86 and 0.97 are observed (47, 58, 70–77).

**Table 2:** **Mean, median, range of scores, number of cases, and Cronbach’ alpha internal consistency of instruments**.

	Mean	SD	Median	Range	Maximum score	Minimum score	Cronbach’s alpha	Number of cases
Mobile Phone Problem Use Scale	68.95	36.89	58.50	234	260	26	0.939	1.126
STAI-S	16.32	9.76	14.00	57	57	0	0.923	1.126
BDI-13	3.60	4.62	2.00	39	39	0	0.877	1.126
STAI-S	16.32	9.76	14.00	57	57	0	0.923	1.126
UPPS-P—total	120.65	24.29	119.00	140	205	65	0.935	1.126
UPPS-P—positive urgency	25.25	9.61	23.00	42	56	14	0.945	1.126
UPPS-P—negative urgency	25.77	7.05	26.00	34	46	12	0.873	1.126
UPPS-P—lack of premeditation	21.70	5.73	22.00	33	44	11	0.868	1.126
UPPS-P—lack of perseverance	20.19	4.93	20.00	26	36	10	0.800	1.126
UPPS-P—sensation seeking	27.73	8.33	28.00	36	48	12	0.889	1.126
AUDIT alcohol	6.60	3.79	6.00	26	27	1	0.595	909

*Mean, SD, median, range, maximum and minimum score, Cronbach’s alpha, and number of cases per scale*.

Similarly, with the UPPS-P, a Cronbach’s alpha of 0.935 was obtained, with an average score of 120.65 and an SD of 24.29 when considering the scale as a whole. Regarding its individual dimensions, for negative urgency, the alpha is 0.873 (Mean = 25.77, SD = 7.05); for lack of premeditation, it is 0.868 (Mean = 21.70, SD = 5.73); for lack of perseverance, it is 0.800 (Mean = 20.19, SD = 4.93); for sensation seeking, it is 0.889 (Mean = 27.73, SD = 8.33); and for positive urgency, it is 0.945 (Mean = 25.25, SD = 9, 61) (Table 2). These data are very consistent with studies such as the Spanish adaptation by Verdejo-García et al. (66), who obtain an alpha of 0.94 for the total scale, 0.87 for negative urgency, 0.87 for lack of premeditation, 0.79 for lack of perseverance, 0.89 for sensation seeking, and 0.93 for positive urgency.

Regarding the BDI-13 and the STAI-S, historically, their internal consistency is strong. Thus, in the BDI-13, a range of Cronbach’s alpha coefficients of 0.78–0.97 has been found (78), with 0.877 in our research, a mean score of 3.60, and an SD of 4.62. The same can be said of the STAI-S, with a historical outcome of internal consistency in Spain of between 0.80 and 0.94 (79, 80). In our case, it is 0.923, with a mean score of 16.32 and an SD of 9.76 (Table 2).

In the case of the AUDIT, several studies in Spain have given it an adequate reliability, validity, and sensitivity in hospital and primary health-care clinical populations (81–83), with Cronbach’s alpha coefficients of between 0.81 and 0.93. However, in our study, the alpha value is weak, coming to 0.595, a mean score of 6.60 and an SD of 3.79 (Table 2). However, Contel et al. (84) obtain a Cronbach’s alpha coefficient of 0.62 in a comparative study among non-alcoholic patients.

Much the same has occurred with the FTND, which is designed to detect heavy smokers at risk of disease. In general, various studies show a low and variable internal consistency for the FTND, with Cronbach’s alpha coefficients of 0.61 (65), 0.83 (85), and 0.66 in its Spanish adaptation (86). In our sample, it is very low, 0.268, with an average score of 2.35 and an SD of 2.41.

Both results may be because they are instruments with few items, especially in the case of the FTND, designed to work with clinical populations versus our general population survey.

### Relationships with and Influence in Regard to Problematic Mobile Phone Use

Therefore, we finally considered the overall sum of the scores of the state anxiety scale (STAI-S), depression inventory (BDI-13), alcohol use test (AUDIT), global impulsivity and impulsivity in each of its five dimensions (UPPS-P), and problematic mobile phone use (MPPUS), both in its total score and with its three factors.

Our hypothesis considers that only anxiety and impulsivity may have a predictive power in regard to problematic mobile phone use. For this reason, the analyses consider, on the one hand, a Pearson’s correlation matrix to determine the relationships of these variables with problematic mobile phone use and, on the other hand, a multiple regression analysis to ascertain which actually predicts it.

This aims to determine which variables are predictors and which coexist and are related to problematic mobile phone use that may, in this case, also result from it.

### Relationship with Problematic Mobile Phone Use and between Variables

In general, anxiety and impulsivity in its five dimensions, especially positive and negative urgency, have a stronger relationship with problematic mobile phone use. To a lesser extent, alcohol use and depression are also significantly related.

When considering the three factors of the MPPUS, anxiety is significantly related to abuse and dependence and to craving and loss of control. The total impulsivity may significantly correlate with the three factors of the MPPUS but mainly with the first two, in which positive urgency may primarily be related to abuse and dependence whereas negative urgency may be present in both. Lack of premeditation and lack of perseverance also show a higher correlation with abuse and dependence, although to a lesser extent.

Alcohol use correlates with the three factors, with a higher correlation in Abuse and Dependence, whereas depression has the greatest correlation with craving and loss of control.

Outside problematic mobile phone use and when analyzing the relationships between variables, we observe that alcohol use is related to anxiety and total impulsivity in all its dimensions and, to a lesser extent to depression. In turn, depression may maintain important relationships with anxiety and impulsivity, mainly through negative urgency and lack of perseverance (Table 3).

**Table 3:** **Pearson correlation coefficients between the total score of the Mobile Phone Problem Use Scale (MPPUS), its three factors, and psychological variables, and alcohol use**.

	Problematic use MPPUS—total	Abuse and dependence (factor I)	Craving and loss of control (factor II)	Social environment dependence (factor III)
BDI-13 depression	0.253**	0.147**	0.174**	0.110**
STAI-S—anxiety	0.434**	0.342**	0.281**	0.095**
AUDIT alcohol	0.265**	0.209**	0.121**	0.116**
UPPS-P—impulsivity total	0.426**	0.353**	0.243**	0.117**
Negative urgency	0.375**	0.209**	0.256**	0.187**
Positive urgency	0.385**	0.346**	0.179**	0.120**
Lack of perseverance	0.269**	0.258**	0.198**	0023
Lack of premeditation	0.209**	0.255**	0.067*	0.011
Sensation seeking	0.178**	0.123**	0.121**	0.051
**Pearson correlation coefficients between psychological variables and alcohol use**				

	**Depression BDI-13**	**Anxiety STAI-S**	**Alcohol AUDIT**	**Impulsivity UPPS-P**

BDI-13 depression	–	0.498**	0.173*	0.237**
STAI-S—anxiety	0.498**	–	0.207**	0.376**
AUDIT alcohol	0.173*	0.207**	–	0.206**
UPPS-P—total impulsivity	0.237**	0.376**	0.206**	–
Negative urgency	0.308**	0.383**	0.121**	0.799**
Positive urgency	0.192**	0.350**	0.150**	0.865**
Lack of premeditation	0.095**	0.157**	0.120**	0.484**
Lack of perseverance	0.266**	0.325**	0.152**	0.544**
Sensation seeking	−0.014	0.068*	0.148**	0.587**

*Probability of significance of the Pearson correlation coefficients for the value 0.05 (*p* ≤ 0.05).

**Probability of significance of the Pearson correlation coefficients for the value 0.01 (*p* ≤ 0.01).

### Regression Analysis of Problematic Mobile Phone Use

The predictive value of state anxiety, depression, impulsivity in its five dimensions, and alcohol use was analyzed by considering problematic use in the MPPUS as a dependent variable. Overall, these variables explain 28.7% of total problematic mobile phone use, 21.3% of abuse and dependence (factor I), 10.9% of craving and loss of control (factor II), and 6.5% of social environment dependence (factor III).

Specifically, in total problematic mobile phone use, anxiety and alcohol use may be the variables with the greatest explanatory power, in addition to impulsivity expressed in positive urgency and, to a lesser extent, negative urgency.

At the same time, the abuse and dependence factor, in addition to alcohol use and anxiety, may be determined by positive urgency and lack of premeditation, whereas the craving and loss of control factor, in addition to anxiety, negative urgency, and lack of perseverance, have relevance. Finally, in the social environment dependence factor together with alcohol use, negative urgency is maintained with lack of perseverance, which is interpreted as insistence or positive perseverance when it has a negative value (Table 4).

**Table 4:** **Multiple regression analysis of the total score and factors of the mobile phone problem use scale (MPPUS)**.

	**Total MPPUS**			**Abuse and dependence (F-I)**			**Craving and loss of control (F-II)**			**Social environment dependence (F-III)**		
Adjusted *R*-squared	0.287 (*F* = 46.733. *p* = 0.000)			0.213 (*F* = 31.696. *p* = 0.000)			0.109 (*F* = 14.881. *p* = 0.000)			0.065 (*F* = 8.826. *p* = 0.000)		
	**β**	***t***	***p***	**β**	***t***	***p***	**β**	***T***	***p***	**β**	***t***	***p***

Alcohol—AUDIT	0.155	5.288	0.000	0.109	3.552	0.000	0.053	1.609	0.108	0.106	3.162	0.002
Depression—BDI-13	0.010	0.308	0.758	−0.013	−0.376	0.707	−0.006	−0.168	0.867	0.036	0.935	0.350
Anxiety—STAI-S	0.273	7.787	0.000	0.250	6.800	0.000	0.181	4.612	0.000	0.007	0.178	0.859
Negative U—UPPS-P	0.121	2.748	0.006	−0.193	−4.165	0.000	0.208	4.216	0.000	0.253	4.993	0.000
L. Premeditation—UPPS-P	0.060	1.718	0.086	0.122	3.336	0.001	−0.050	−1.290	0.197	0.018	0.462	0.644
L. Perseverance—UPPS-P	0.006	0.165	0.869	0.031	0.795	0.427	0.108	2.595	0.010	−0.155	−3.612	0.000
Sensation S—UPPS-P	0.043	1.367	0.172	0.032	0.974	0.331	0.052	1.481	0.139	−0.028	−0.785	0.433
Positive U—UPPS -P	0.154	3.391	0.001	0.330	6.885	0.000	−0.076	−1.495	0.135	−0.021	−0.397	0.692

*Adjusted R^2^, β values, and t-statistic, with probabilities where a significance of 5% was considered the maximum*.

Therefore, despite our initial hypothesis, which only considered anxiety and impulsivity as predictor variables, we observe that, in addition to them, alcohol use also explains problematic mobile phone use. However, and as expected, although it has a connection with depression, it has no explanatory power in this research, it is not a direct cause, and it may be the result or mediator of problematic use.

## Discussion

We have analyzed depression, state anxiety, impulsivity, and alcohol use, both for their direct relationships and for their predictive power in regard to problematic mobile phone use at large, and specifically in regard to the three factors of the MPPUS that were considered (Abuse and Dependence, Craving and Loss of Control, and Social Environment Dependence).

Our hypothesis was based on the fact that although there is clear proof in the scientific literature that these variables are related to problematic mobile phone use, only anxiety and impulsivity may actually be predictive of it. In this regard, the rest may be considered effects or mediators.

Initially, based on the interrelationships among all variables, anxiety and impulsivity in its five dimensions, especially positive and negative urgency, are the factors that have stronger relationships with problematic mobile phone use. To a lesser extent, alcohol use and depression also have significant relationships. Regarding the MPPUS factors, anxiety and impulsivity, expressed in terms of positive and negative urgency, maintain the highest relationships with the factors of abuse and dependence and craving and loss of control. The dimensions of lack of perseverance and lack of premeditation may also maintain their closest relationships with abuse and dependence.

Moreover, in considering the joint predictive value of anxiety, depression, impulsivity in its five dimensions, and alcohol use as independent variables, we note that they eventually explain problematic mobile phone use in 28.7% of all cases, with 21.3% for factor I, abuse and dependence, 10.9% for factor II, craving and loss of control, and 6.5% for factor III, social environment dependence.

Specifically, anxiety, alcohol, and impulsivity expressed through positive and negative urgency have a relevant predictive weight. Put differently, the factor analysis may help explain this result. It shows that, in abuse and dependence, positive urgency predominates, motivated by precipitation derived from positive affective states, in addition to lack of premeditation or reflection on the consequences, that is, a determined impulse to use mobile phones due to anxiety, leading to an impulsive behavior resulting from pleasant affective states in which alcohol may have an important presence. Only when we observed craving and loss of control did we find that mobile phones, in this case through negative urgency, also represent a means of escaping from dysphoric states with anxiety and lack of perseverance. Although social environment dependence is a factor that is little explained, it shows that the use of mobile phones as an escape from unpleasant emotional states leads to behavioral persistence, most likely social contact seeking, and that these feelings go together with alcohol use.

However, the assessment of tobacco use was finally unhelpful in this research. This result most likely has more to do with the choice of the measuring instrument than with the variable itself. We have already observed that, historically, the FTND was not an instrument with appropriate coefficients of internal consistency, which is an aspect that was found in this study. Clearly, the FNTD was designed for a rapid clinical assessment, with few items in the detection of smokers with high nicotine dependence. This makes it inadequate for our general population sample, with 23.1% of smokers and mean scores (M = 2.35, SD = 2.41) that are well below the minimum requirements (≥4 points) of dependence in this test. However, not all studies have found consistent results with problematic mobile phone use. In our case, we cannot confirm that it coexists or is a predictor, but we genuinely believe the existence of some type of relation.

Regarding depression, according to our initial hypothesis, it has a relationship with problematic mobile phone use but with no final predictive power. Similarly, it has been historically investigated in relation to different types of behavioral addictions, also coexisting with alcohol and other drugs (41). However, in the case of mobile phones, Ghasempour and Mahmoodi-Aghdam (87) find that depression was able to predict addiction by using mediator variables such as feelings of inferiority and low self-esteem, which may lead to seeking secure relationships through messages and the use of social networks. Babadi-Akashe et al. (88) also find a relationship among depression, obsessive-compulsive disorders, and interpersonal sensitivity among users with mobile phone dependence. Augner and Hacker (89) show significant relationships among mobile phone abuse, chronic stress, emotional stability, and depression among young women. Tavakolizadeh et al. (90) observe a coexistence between the tendency toward somatization, anxiety, and depression and excessive mobile phone use. Giota and Kleftaras (91) find that the problematic use of social networks was related to, among other aspects, neuroticism and depression, especially among women. Chen (92), Toda and Ezoe (93), and Kim et al. (94) confirm the relationship between depression and mobile phone addiction, which is a means of relieving or balancing negative affective states. However, opposite results have also been reported, as in the case of Whiteside and Lynam (61), who do not find such a relationship.

Depression is also related to impulsivity; however, only the latter may have predictive power in regard to problematic use, as was observed above. In this sense, Smetaniuk (1) finds that age, depression, extraversion, and low impulse control are significant predictors of problematic mobile phone use. In the case of impulsivity, as in our research, various studies have shown its relationship with mobile phone addiction and dependence (29), particularly due to the dimensions of attentional impulsivity (31), lack of perseverance, and negative urgency (21, 95). Mottram and Fleming (96) find that impulsivity and particularly lack of perseverance may be predictors of compensatory social behaviors, such as online interactive activities or social networks. Roberts and Pirog (97) also find that materialism, or the tendency to want and have expensive products or the most prestigious brands, and impulsivity predict mobile phone addiction, especially through text messages. Walther et al. (6) also indicate that impulsivity lies at the root of behavioral addictions, specifically gamblers and problematic video gamers, and in these cases, there is a coexistence with alcohol, tobacco, and cannabis use.

We have also observed that alcohol use has a significant predictive weight. Kuss and Griffiths (98) have already indicated its coexistence with social networking abuse; in turn, however, alcohol use has a significant relationship with impulsivity. This is also found, both in this research and in other studies, in which it is observed that lack of control or response inhibition (39), positive urgency (37), and negative urgency linked to lack of perseverance (36, 38) may be involved. Simultaneously, impulsivity may be a direct predictor of alcohol dependence, especially from the perspective of negative urgency, lack of perseverance, and lack of premeditation as a means of alleviating negative affective states (99). Impulsive behavior is also linked to tobacco use, which we could not observe in this research, specifically from negative urgency, which is a predictor of craving (95). In the same vein with adolescents, Gunnarsson et al. (100) note the relationship between impulsivity and antagonism with the environment and tobacco and alcohol use. Malmberg et al. (101) also find a coexistence between personality factors and substance use in which this type of consumption can also determine certain personality traits, such as impulsivity in general and sensation seeking in particular, which are factors of greater interaction with tobacco and alcohol use.

Therefore, as observed in this research, the weight and significance of anxiety in the context of addictions is known, for example, in the case of social anxiety that leads to social environment dependence. Social anxiety predicts mobile phone use based on an increased use of text messages (61, 102) in which variables such as the perception of self-efficacy and self-worth seem to intervene (103). One of its expressions would be “textiety” or the anxiety over receiving and immediately responding to text messages (104), with social networks as a means of seeking support and safety (105); here, imitating others and low self-esteem become important (106).

Additionally, very much in line with our results, Lee et al. (107) find that the compulsive use of smartphones may be related to social anxiety. Mobile phones would minimize the perceived risks and personal insecurity in relation to the environment. Similarly, Bian and Leung (108) show that social anxiety, shyness, and loneliness may consequently increase the likelihood of smartphone addiction. Hong et al. (109) also find that social extraversion and anxiety are related to mobile phone addiction, in which it can be a means of reducing or balancing that anxiety.

In short, and as stated above, our initial hypothesis is based on the fact that, although there are obvious and proven relationships among the variables that we consider, only anxiety and impulsivity may be able to predict problematic mobile phone use. However, the results show that, in addition to these, alcohol use may also have predictive power and, in this case, depression may remain a mediator variable, resulting from or coexisting with this problematic use.

It must also be noted that the use of the MPPUS factors, in addition to the overall result, has also made it possible to differentiate the components of problematic use, providing a further specification of the variables with the predictive power for each and, therefore, obtaining a better profile of mobile phone dependence. Despite the use of three of these factors, we note that the best predictive values are concentrated in the first two. Additionally, a cautious approach should be taken in regard to the social environment dependence factor because it is the worst explained by the regression model.

However, it is clear that the variables that were considered as independent variables may also play a mediating role or be dependent on problematic mobile phone use. As shown by other studies, there is not a unique direction, and methodological approaches can be diverse, with a large environment of interrelations among the variables.

Finally, is important to emphasize that the cell phone use is clearly not an extension of computer or Internet use. Both, Internet and cell phone show some differences: cell phone abuse responds to a pattern of greater lack of impulse control (30), while depression appears to be more consubstantial with problematic Internet use. In the same way, anxiety seems to be more consubstantial with problematic cell phone use, specifically in the context of social environment *via* text messaging (45). In any case, further research is necessary to clearly establish the contribution of impulsivity to problematic cell phone use, by using more adequate neuropsychological tests. If confirmed, impulsive patients might need specific advisory interventions for diminishing the risk of developing a problematic mobile phone use.

## Conclusion

Our results on problematic mobile phone use in Spain are in line with other studies concerning various types of addictive behaviors, with substances such as alcohol or pathological gambling. This similarity and comparability with recognized addictions show that, behind problematic mobile phone use, there is an almost identical structure of the variables and interrelationships that, comparatively, may speak in favor of considering mobile phone use to be a behavioral addiction. Therefore, and given the demonstrated relationship between impulsivity not only with drugs use but also with other type of behavioral addictions as the mobile phone use, it could be important to prevent the problematic phone use considering the impulsive personality trait. These preventive actions could essentially affect to adolescents being important also to consider a broader ranges of population.

### Limitations of This Research

Sociodemographic and drug use differences among the problematic phone users have not been considered in this study. At the same time, the assessment of impulsivity and other variables with subjective methods as questionnaires present important limitations. Next steps and future research will provided specific analysis in this sense as well as incorporate computerized tasks in the impulsive behavior research.

## Ethics Statement

The present project was approved by the Ethics Committee of i+12 Institute in Madrid. The study is a web platform-based survey with includes an inform consent at the beginning of the questionnaire where the participants are informed of the purpose of the study. All subjects gave their free acceptance in accordance with the Declaration of Helsinki. All the procedures guarantee the generation of completely anonymized datasets.

## Author Contributions

FF and GR designed the study with the help of JD-S. JD-S did the survey, collected the data, and prepared statistical files. HT did the statistically analysis. First draft of manuscript was written by JD-S. The draft was finally reviewed in depth by all authors. Financial support was obtained by FF.

## Conflict of Interest Statement

The authors declare that the research was conducted in the absence of any commercial or financial relationships that could be construed as a potential conflict of interest.
